# Precision Targeting of Myeloid Cells via Peptide Dendrimer‐Lipid Nanocarriers: A Novel Platform for Potent Cancer Immunotherapies

**DOI:** 10.1002/advs.202514417

**Published:** 2025-09-19

**Authors:** Lawrence James Billing, Liam Martin, Tristan Henser‐Brownhill, Parisa Samangouei, Adam Leach, Phoebe Knight, Marina Apostolidou, Aaqib Ladak, Benita Nagel, Albert Kwok

**Affiliations:** ^1^ Nuntius Therapeutics Limited London W10 5JJ UK

**Keywords:** immuno‐oncology, immunotherapy, mRNA vaccines, nanomedicine, RNA therapies

## Abstract

Selective targeting of myeloid cells (MCs) holds therapeutic potential as an immuno‐oncology approach. However, MC targeting using existing gene delivery platforms faces many barriers to clinical use, such as production scalability and off‐target delivery to the liver and non‐MCs. Here, a novel peptide dendrimer‐lipid (PDL) nanocarrier for systemic administration, which overcomes many of these barriers, is characterized. This MC targeting nanocarrier (MCTN) is self‐assembling, de‐targets the liver, and selectively targets the spleen and MCs within the tumor microenvironment (TME). MCTN is validated as a cancer vaccine platform utilizing full‐length antigens, generating robust immune responses with broad HLA‐haplotype coverage. Finally, using a payload encoding constitutively active interferon regulatory factor 5 (IRF5), MCTN is found to suppress solid tumor growth and modulate the TME by improving CD8+ T‐cell infiltration while depleting immunosuppressive cells.

## Graphical abstract

Precision targeting of the spleen and myeloid cells of the tumor microenvironment, with minimal hepatic delivery, is achieved using a modular peptide dendrimer‐lipid nanocarrier. Functional assays, transcriptomics, and rodent models validate this novel myeloid cell targeting nanocarrier as an efficacious immunotherapy, both as a cancer vaccine platform and tumor microenvironment immunomodulator.

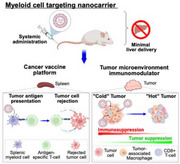

## 1 Introduction

Myeloid cells (MCs) consist of immune cell types such as dendritic cells and macrophages, which perform critical functions including tissue repair, innate immune responses, and initiating adaptive immune responses through antigen presentation and cytokine release.^[^ ^1^ ^]^ Dysregulation and malfunction of MCs contribute to a diverse range of diseases, including cancer and autoimmune diseases.^[^ ^1^, ^2^ ^]^ Exploiting the ability of MCs to present antigens to the adaptive immune system, cancer vaccines have been developed based on mRNA delivery to antigen‐presenting cells (APCs) using lipoplexes or lipid nanoparticles (LNPs) for a variety of solid tumors, including melanoma, pancreatic cancer, and non‐small cell lung cancer.^[^ ^3^ ^]^ One example is the “off‐the‐shelf” vaccine BNT116, an RNA‐lipoplex (LPX) vaccine administrated intravenously to target splenic APCs. BNT116 is currently in phase 2 testing for treating advanced non‐small cell lung cancer.^[^ ^4^ ^]^ Immuno‐oncology therapies are also being developed to directly target MCs within the tumor microenvironment (TME). Within the TME, tumor‐associated macrophages (TAMs) assist tumor expansion, supporting angiogenesis and tumor cell motility while creating a highly immunosuppressive, immunologically “cold” microenvironment. This immunosuppressive environment impedes the entry and function of tumor‐infiltrating lymphocytes (TILs) within the tumor.^[^ ^5^ ^]^ Activating TAMs can reverse this phenotype, leading to tumor growth reduction.^[^ ^6^ ^]^ Although MCs show potential as a target for nucleic acid‐based cancer therapy, they are difficult to transfect and to selectively target in vivo using traditional gene delivery platforms such as clinically approved viral vectors and LNPs.^[^ ^7^ ^–^ ^12^ ^]^

Previously, we demonstrated that our peptide dendrimer‐lipid (PDL) nanocarrier platform could outperform state‐of‐the‐art LPX in terms of transfection with a favorable toxicity profile.^[^ ^13^ ^]^ Here, we developed and validated a self‐assembling PDL‐based nanocarrier for selective MC targeting (MCTN). We established that MCTN efficiently transfects both murine and human macrophages and systemic administration delivers nucleic acids to the spleen, while, unlike canonical LNPs, de‐targeting the liver. When compared with dose‐matched LPX, MCTN yielded superior payload delivery to the spleen following systemic administration. Consequently, we validated our MCTN platform as a novel cancer vaccine delivery modality by demonstrating in vivo induction of antigen‐specific T‐cell responses and suppression of tumor growth in a tumor model. Finally, we demonstrated that systemic administration of MCTN can be used to deliver payloads directly to the TME, selectively targeting MCs over other cell types. Utilizing encapsulated mRNA encoding the proinflammatory transcription factor interferon regulatory factor 5 (IRF5), we demonstrated the immunomodulatory capacity of MCTN, turning the TME immunologically “hot” through depletion of immunosuppressive macrophages while enhancing CD8+ T‐cell infiltration. In parallel with these modifications to the TME, we observed a suppression in tumor growth, highlighting the therapeutic potential of targeting the TME using MCTN.

## 2 Results

### 2.1 MCTN Efficiently Transfects Macrophages

**Figure 1:**
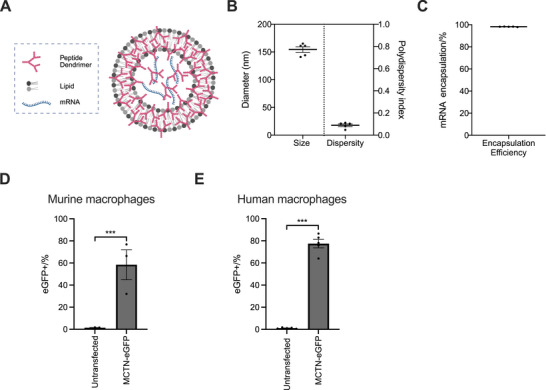
Macrophage transfection efficiency of MCTN. A) Schematic illustration of self‐assembling MCTN comprised of peptide dendrimer, lipid, and mRNA payload. B) Average size, dispersity, and (C) mRNA encapsulation efficiency of MCTN. *n* = 5 independent batches. D) Murine bone marrow‐derived macrophages (*n* = 3 biologically independent samples per group) and (E) human monocyte‐derived macrophages (CD14+CD11b+; *n* = 5 donors per group) were transfected overnight with MCTN‐eGFP and analyzed by flow cytometry. Bars/lines indicate mean values ± SEM in B, C, D, and E. Two‐tailed ratio paired t‐tests were used to examine statistical differences for (D & E). ^***^ *p* < 0.001.

We evaluated the transfection efficiency of MCTN (see Figure S1, Supporting Information for the structural details of the peptide dendrimer and lipid components of MCTN) through flow cytometry analysis of primary murine bone marrow‐derived macrophages (BMDMs) and human monocyte‐derived macrophages (huMDM) transfected overnight with MCTN encapsulating eGFP mRNA (MCTN‐eGFP; **Figure** **1**A). MCTN‐eGFP formulations had an average size of 154 nm, an average polydispersity index (PDI) of 0.090, and a high average mRNA encapsulation efficiency with 98.2% (Figure 1B,C). MCTN‐eGFP transfected an average of 58.4% of murine BMDMs (Figure 1D; Figure S2, Supporting Information) and 77.5% of huMDM (Figure 1E; Figure S3, Supporting Information).

Macrophage uptake of MCTN largely occurs via phagocytosis and macropinocytosis (Figure S4A, Supporting Information). Similarly, using the same set of inhibitors, phagocytosis and macropinocytosis have previously been found as key mediators of MC uptake of LPX formulations.^[^ ^14^ ^]^ Macrophage targeting by MCTN could be attributed to the many arginine residues in MCTN particles, which can stimulate macropinocytosis and phagocytosis.^[^ ^15^, ^16^ ^]^ Macropinocytosis and phagocytosis are also distinguishing functions of MCs, and their involvement in MCTN uptake provides a potential mechanism for MC selectivity over other cell types.^[^ ^17^ ^]^ Endosomal acidification has previously been identified as a key mechanism underlying endosomal escape for mRNA encapsulated by LNP. Our results, using the V‐ATPase inhibitor bafilomycin A1, suggest an analogous role of endosomal acidification in MCTN‐mediated mRNA delivery (Figure S4A, Supporting Information).^[^ ^18^, ^19^ ^]^

Dose‐matched experiments examining the transfection efficiencies of MCTN using murine macrophages (J774 cells) suggest MCTN transfects macrophages much more efficiently than both LNPs based on the Moderna COVID‐19 vaccine and the LPX system (Figure S4B, Supporting Information). Our results suggest MCTN yields superior macrophage transfection efficiency compared with other leading nanocarrier systems and possibly also outperforms mannosylated nanoparticles and F4/80 antibody conjugated LNPs, which have previously been found with lower transfection efficiencies in vitro of ≈30% and 40% of macrophages, respectively.^[^ ^20^, ^21^ ^]^

### 2.2 MCTN Delivers Payloads to the Spleen and De‐Targets the Liver

**Figure 2:**
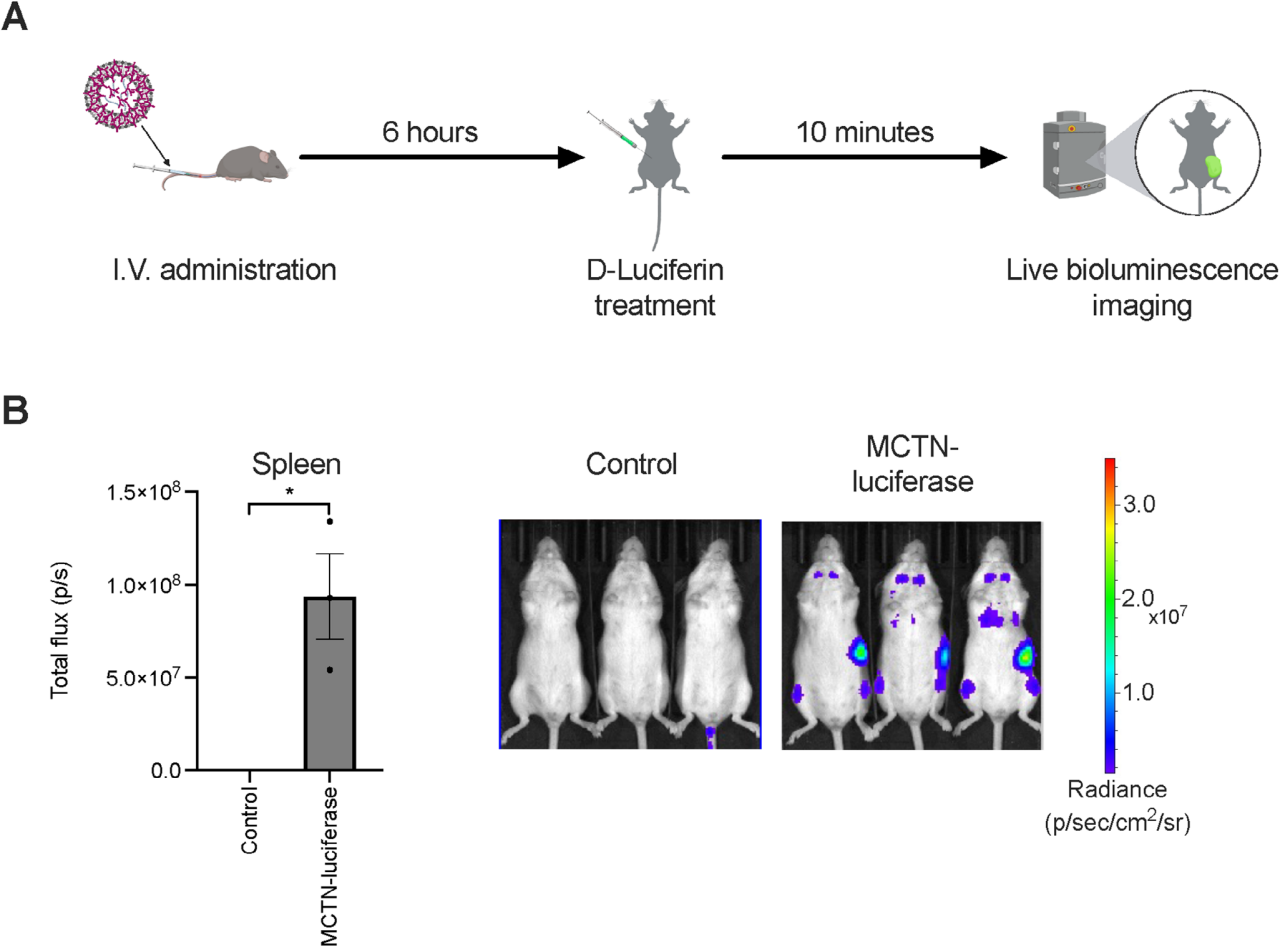
Biodistribution of MCTN following intravenous (I.V.) administration in CD‐1 mice was examined using luciferase encoding mRNA. A) Schematic overview of the experimental timeline. B) In vivo bioluminescence measurements for the spleen from control and MCTN‐luciferase‐treated mice. Bioluminescence images of live mice treated with unencapsulated luciferase mRNA (control) or MCTN encapsulated luciferase mRNA (MCTN‐luciferase) are shown on the right. *n* = 3 mice per group. Bars indicate mean values ± SEM. Statistics evaluated by two‐tailed unpaired *t*‐tests. ^*^ *p* < 0.05.

We examined the in vivo biodistribution profile of MCTN through bioluminescence analysis of animals dosed systemically with MCTN encapsulating mRNA encoding luciferase (MCTN‐luciferase; **Figure** **2**A). The in vivo luciferase signal in the spleen was significantly higher in the MCTN‐luciferase group compared with the control group (Figure 2B), while the signal in the liver was undetectable. Ex vivo bioluminescence analysis of isolated livers and spleens from MCTN‐luciferase‐treated mice similarly identified a strong luciferase signal in the spleen and, conversely minimal luciferase signal in the liver (Figure S5A, Supporting Information). Therefore, MCTN overcomes a major drawback of canonical LNPs, namely preferential targeting of the liver, enabling intravenous delivery of therapeutic transgenes to the spleen without off‐target impact on the liver.^[^ ^8^, ^22^ ^]^

Systemic delivery of mRNA to splenic APCs using the LPX delivery platform elicits stronger immune responses than localized delivery, mediating tumor rejection and enabling treatment with low doses.^[^ ^14^ ^]^ Indeed, the LPX vaccine autogene cevumeran is administered systemically at low doses, such as 25 µg in clinical trials, while the intramuscularly administered mRNA‐4157 (LNP‐based) is dosed at a higher dose of 1 mg in patients.^[^ ^23^, ^24^ ^]^ When we compared splenic delivery of luciferase mRNA by MCTN to dose‐matched LPX we found that the luciferase signal of the spleen was on average 343‐fold greater for the MCTN (Figure S5B, Supporting Information). Using flow cytometry analysis, we also examined the cell types within the spleen that were targeted in vivo by MCTN through administration of Alexa Fluor 488‐tagged mRNA encapsulated by MCTN. MCTN preferentially targeted monocytes, macrophages, and dendritic cells over lymphocytes (Figures S6 and S7, Supporting Information).

### 2.3 Systemic MCTN is an Efficacious Cancer Vaccine Delivery Modality

Given that MCTN efficiently delivers mRNA to MCs in the spleen, a key site for antigen presentation, we examined the potential of MCTN as a vaccine delivery system.^[^ ^25^ ^]^ We utilized the chicken protein ovalbumin (OVA), an antigen routinely used to assess vaccine delivery platform efficacy, including cell, peptide, DNA, viral, and other mRNA‐based platforms, in mice.^[^ ^26^, ^27^, ^28^, ^29^ ^]^ We used unmodified mRNA encoding full‐length OVA encapsulated by MCTN (MCTN‐OVA).

**Figure 3:**
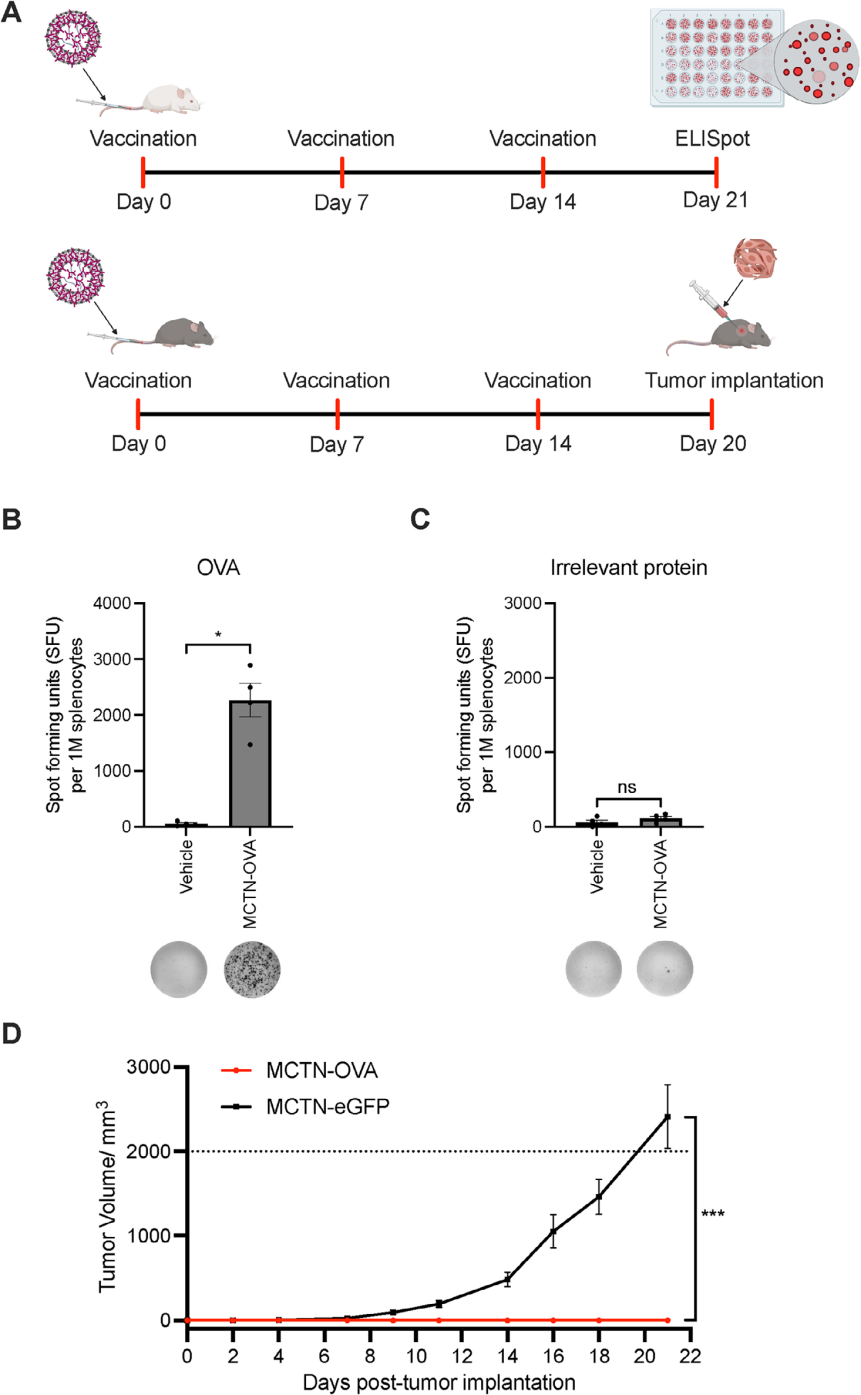
MCTN‐OVA treatment generates immune responses and tumor rejection. A) Timelines indicating vaccination schedule with the ELISpot experiment indicated on top and the prophylactic tumor model on the bottom. B,C) IFN‐γ ELISpot assays run on splenocytes isolated from Balb/c mice vaccinated with MCTN‐OVA or administered with vehicle. Example images of ELISpot wells are shown below each condition. *n* = 4 mice per group. Bars indicate mean values ± SEM. Statistics were evaluated using two‐tailed Mann‐Whitney tests. D) B16F10‐OVA tumor growth in C57BL6 mice prophylactically vaccinated with MCTN‐OVA or MCTN‐eGFP (control). The dotted line indicates the humane endpoint in terms of tumor size (2000 mm^3^). *n* = 15 mice per group. Plotted values reflect mean measurements ± SEM. A two‐way mixed‐effects model with Geisser‐Greenhouse correction was used to evaluate statistical significance between treatment groups. ^*^ *p* < 0.05, ^***^ *p* < 0.001 and non‐significant (ns) results *p* > 0.05.

We first examined the ability of MCTN‐OVA to induce in vivo T‐cell immune responses by IFN‐γ ELISpot assays (**Figure** **3**A). Splenocytes isolated from mice vaccinated with MCTN‐OVA produced robust and significantly higher spot counts than OVA responses from the vehicle group (Figure 3B; Figure S8, Supporting Information). Stimulation of splenocytes with an irrelevant protein did not induce a significant difference in the number of spots in the MCTN‐OVA vaccinated group compared with the vehicle group (Figure 3C). These results indicate that our MCTN‐based vaccine can significantly induce antigen‐specific T‐cell responses and serve as an mRNA vaccine delivery platform.

When examining the treatment efficacy of our vaccine platform in an OVA‐expressing B16F10 tumor model, we observed that MCTN‐OVA vaccination effectively abated tumor growth compared with the control group (Figure 3D). 12 out of 15 mice in the control group reached a tumor size of 2000 mm^3^ by 21 days, while none of the mice in the MCTN‐OVA vaccinated group had any tumor. Previously, cancer vaccines delivered via different vectors, including plasmid DNA, adenoviral, and peptide vaccines, have shown therapeutic potential both in preclinical and clinical studies against a range of solid tumors.^[^ ^3^, ^14^, ^24^, ^28^, ^30^, ^31^, ^32^, ^33^ ^]^ However, when comparing these systems in terms of efficacy in suppressing tumor growth using the B16F10 model, tumors were observed by day 15 post‐tumor implantation using all these delivery modalities except for the mRNA‐based approaches.^[^ ^14^, ^28^, ^30^, ^33^ ^]^ As previously discussed, MCTN exhibits superior splenic delivery of mRNA compared with the state‐of‐the‐art lipoplex system (Figure S5B, Supporting Information). Together, these results suggest that MCTN may provide a more potent cancer vaccine modality than existing platforms.

### 2.4 MCTN can Deliver Payloads Selectively to MCs Within the Tumor Microenvironment

**Figure 4:**
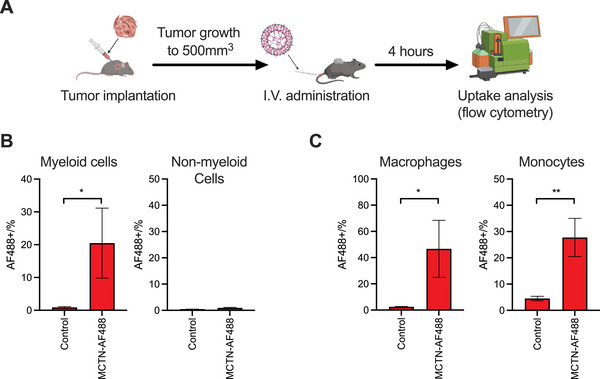
A) Schematic detailing key stages of the uptake study. Tumor uptake of Alexa Fluor 488 (AF488) tagged mRNA following intravenous (I.V.) treatment with MCTN encapsulated AF488‐mRNA (MCTN‐AF488) or unencapsulated AF88‐mRNA (control) was detected by flow cytometry. B) AF488‐mRNA uptake by MCs (CD45+CD11b+) and non‐MCs. C) AF488‐mRNA uptake by tumor‐associated macrophages (CD45+CD11b+CD11c‐Ly6G‐Ly6C‐F4/80+) and proinflammatory monocytes (CD45+CD11b+CD11c‐Ly6G‐Ly6C+). *n* = 3 mice per group. Bars indicate mean values ± SEM. Unpaired two‐tailed *t*‐tests are used to evaluate statistics. ^**^ *p* < 0.01, ^*^ *p* < 0.05.

Within the TME, MCs such as TAMs can induce immunosuppression and support tumor growth. Targeting MCs within the TME presents an immunotherapy opportunity to remodulate the TME from an immunosuppressive “cold” state into a “hot” state, marked by reduced immunosuppression, depletion of pro‐tumor cells such as TAMs, and increased infiltration of anti‐tumor effector cells like CD8+ T‐cells.^[^ ^34^ ^]^ To demonstrate that MCTN can target the TME, we examined the biodistribution of MCTN‐luciferase in subcutaneous MC38 tumor‐burdened C57BL6 mice (Figure S10, Supporting Information). The MC38 syngeneic model is a colorectal tumor model that exhibits a high number of immunosuppressive MCs within the TME.^[^ ^35^, ^36^ ^]^ Treatment with MCTN‐luciferase yields higher luciferase activity within the tumor region compared with the control group (Figure S10B, Supporting Information). We then examined the uptake of MCTN by MCs within the TME using flow cytometry analysis of dissociated MC38 tumors (**Figure** **4**A). Mice were treated intravenously with Alexa Fluor 488‐tagged mRNA (AF488‐mRNA) either encapsulated with MCTN (MCTN‐AF488) or unencapsulated (control). This approach revealed that, on average, MCTN‐AF488 delivered mRNA to 20.5% of the MCs (CD45+CD11b+) within the TME compared with 0.89% of MCs for the control (Figure 4B). Conversely, MCTN‐AF488 delivered mRNA to only 0.93% of the non‐MCs (Figure 4B). We also examined the ability of MCTN to target TAMs (CD45+CD11b+CD11c‐Ly6G‐Ly6C‐F4/80+) and proinflammatory monocytes (CD45+CD11b+CD11c‐Ly6G‐Ly6C+). MCTN‐AF488 delivered mRNA to an average of 46.7% and 27.7% of the TAMs and proinflammatory monocytes, respectively (Figure 4C; Figure S11, Supporting Information). These results indicate that MCTN can deliver payloads directly to the TME, selectively targeting MCs following systemic delivery. Compared with localized delivery methods such as intratumoral delivery, which are technically and logistically challenging to administer, intravenous delivery is simpler to administer using existing outpatient infrastructure and enables targeting of metastatic sites beyond the primary tumor.^[^ ^37^, ^38^ ^]^

### 2.5 MCTN‐IRF5 Transfection Upregulates Anti‐Tumor Genes While Downregulating Pro‐Tumor Genes in Macrophages

Having established that MCTN efficiently transfects MCs within the TME, we then examined the therapeutic potential of this approach by transfecting immunosuppressive BMDMs with mRNA encoding the transcription factor IRF5, which suppresses the pro‐tumor immunosuppressive activities of tumor‐associated macrophages, while upregulating key anti‐tumor proinflammatory pathways.^[^ ^20^, ^39^ ^]^ We modified the amino acid sequence of IRF5 to produce a constitutively active variant through the incorporation of phosphomimetic mutations (S425D, S427D, S430D, and S436D) within the C‐terminal inhibitory domain, improving nuclear translocation and overall protein stability.^[^ ^40^, ^41^ ^]^ Using a constitutively active variant of IRF5 avoids the need for co‐transfection with the IRF5‐activating kinase IKK2 to induce IRF5 nuclear translocation and function.^[^ ^20^, ^42^, ^43^ ^]^

**Figure 5:**
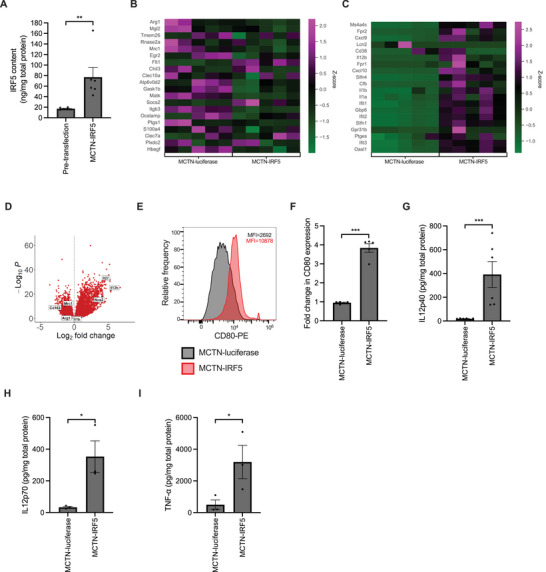
A) IRF5 content for Murine BMDMs transfected with MCTN‐IRF5 for 1 h and lysed 24 h post‐transfection. IRF5 content for each sample was normalized by total protein content. *n* = 6 biologically independent samples. Statistics were evaluated using a two‐tailed ratio paired *t*‐test. ^**^ *p* < 0.01. Heat maps indicating transcript abundance of the top 20 genes expressed in pro‐tumor (B) and anti‐tumor (C) macrophages for MCTN‐luciferase and MCTN‐IRF5‐transfected BMDMs. *n* = 5 biologically independent samples per group. D) Volcano plot of bulk RNA‐seq data displaying the Log_2_ fold change in gene transcript abundance between bone marrow‐derived macrophages transfected with MCTN‐IRF5 and those transfected with MCTN‐luciferase. Statistics were evaluated by DESeq2, which employs the Wald test. *P*‐values were adjusted using the Benjamini‐Hochberg method. Red circles represent genes with fold changes that were statistically significant (*p* < 0.05), while grey circles represent genes with non‐significant changes (*P* > 0.05). E) Relative surface expression of CD80 in BMDM (CD11b+ cells) after overnight transfection with MCTN‐luciferase (control) and MCTN‐IRF5. The histogram shows CD80 surface expression profiles for one example sample of BMDMs transfected with MCTN‐luciferase and MCTN‐IRF5‐transfected cells superimposed. F) Relative CD80 surface expression normalized by the MFI of untransfected control in the CD80 channel. Statistics were evaluated by a two‐tailed unpaired *t*‐test. *n* = 5 biologically independent samples. G) Secretion of IL12 measured from murine BMDMs following transfection with MCTN‐IRF5 or MCTN‐luciferase. *n* = 6 biologically independent samples. IL12 secretion was also measured from transfected human monocyte‐derived macrophages (H), but additionally, TNF‐α content (I) was quantified. *n* = 3 donors. Statistics were evaluated using two‐tailed ratio‐paired *t*‐tests (G–I). ^*^ *p* < 0.05, ^**^ *p* < 0.01, ^***^ *p* < 0.001. For A, F, G, H, and I, bars indicate mean values ± SEM.

Transfection of BMDMs with our IRF5 mRNA construct encapsulated by MCTN (MCTN‐IRF5) was found to increase IRF5 expression by 4.63‐fold on average, indicating effective delivery of the IRF5 payload (**Figure** **5**A). The levels of IRF5 expression induced by our single mRNA payload are in line with those found when BMDMs are co‐transfected with wild‐type IRF5 and IKK2.^[^ ^20^ ^]^ This implies we can achieve similar levels of IRF5 expression using one mRNA payload rather than two.

To examine the effect of MCTN‐IRF5 treatment on the macrophage transcriptome, we applied RNA‐seq analysis to murine BMDMs transfected with MCTN‐IRF5 and MCTN‐luciferase (Figure 5B–D). We found that MCTN‐IRF5 treatment decreased expression of genes associated with pro‐tumor immunosuppressive macrophages and conversely increased expression of genes associated with anti‐tumor macrophages (Figure 5B,C, Supporting Information).^[^ ^44^ ^]^ For example, expression of *Arg1* and *Mrc1*, both key genes associated with pro‐tumor immunosuppressive responses, was downregulated by MCTN‐IRF5 treatment.^[^ ^45^, ^46^ ^]^ In contrast, expression of genes associated with anti‐tumor responses, such as *Il27* and *Il12b*, which encode proinflammatory cytokines and *Nos2* was upregulated by MCTN‐IRF5 treatment (Figure 5D, Supporting Information).^[^ ^47^, ^48^ ^]^ The observed transcriptome changes suggest that MCTN‐IRF5 transfection can shift tumor macrophages from a pro‐tumor state to an anti‐tumor state.

### 2.6 MCTN‐IRF5 Upregulates Surface Levels of T‐Cell Co‐Stimulatory Ligand CD80 and Secretion of Proinflammatory Cytokines

After establishing that treatment with MCTN‐IRF5 shifts the balance in gene expression from pro‐tumor activities toward anti‐tumor activities, we then examined whether this shift had a functional impact. This assessment was made by examining the immunophenotype and cytokine secretion profile of transfected macrophages. BMDMs transfected with MCTN‐IRF5 exhibited a 3.84‐fold increase in CD80 expression (Figure 5E,F; Figure S2, Supporting Information), a key T‐cell co‐stimulatory molecule, significantly higher than MCTN‐luciferase treatment.^[^ ^49^ ^]^ Consistent with the upregulation of *Il12b* expression seen in the RNA‐seq analysis, we found that transfection with MCTN‐IRF5 led to a 22.4‐fold increase in secretion of the anti‐tumor cytokine IL12 (Figure 5G). Similarly, transfection of immunosuppressive huMDM with MCTN‐IRF5 enhanced IL12 secretion, reflected by 11.2‐fold higher levels of IL12p70 compared with the luciferase control, suggesting translatability of our findings to human macrophages (Figure 5H). IL12 is a potent proinflammatory cytokine with a range of different anti‐tumor activities, including activating and increasing the cytotoxicity of T‐cells and inhibiting the activities of immunosuppressive cells within the TME.^[^ ^50^ ^]^ We also found that MCTN‐IRF5 treatment boosted huMDM secretion of proinflammatory TNF‐α by 9.2‐fold (Figure 5I). These findings are consistent with previous studies indicating that IRF5 expression upregulates IL12 and TNF‐α production.^[^ ^20^, ^39^ ^]^

### 2.7 MCTN‐IRF5 Treatment Depletes Immunosuppressive Tumor‐Associated Macrophages While Increasing Cytotoxic CD8+ T‐Cell Infiltration of the Tumor Microenvironment

**Figure 6:**
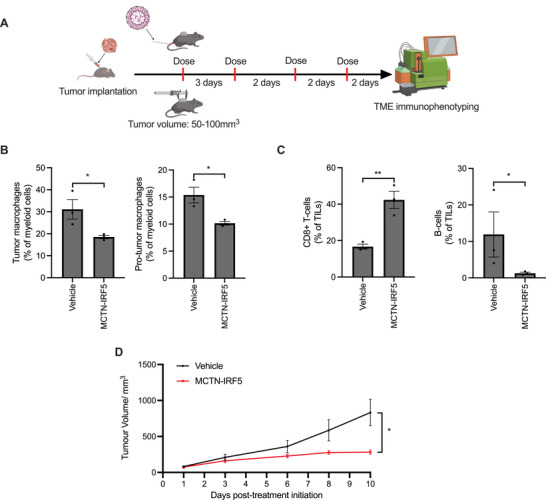
Impact of MCTN‐IRF5 on the tumor microenvironment (TME) and tumor growth in the subcutaneous syngeneic MC38 cancer model. A) Schematic illustrating the treatment schedule and endpoint analysis. B) Summarizes the proportions for total macrophages (CD45+CD11b+Ly6G‐Ly6C‐CD170‐F4/80+) and immunosuppressive pro‐tumor macrophages CD45+CD11b+Ly6G‐Ly6C‐CD170‐F4/80+MHCII‐CD206+) as a fraction of MCs (CD45+CD11b+). C) Summarizes the CD8+ T‐cell (CD45+CD3+CD8+) and B‐cell (CD45+CD19+) fractions of tumor‐infiltrating lymphocytes (CD45+; TILs). *n* = 3 mice per group. D) Subcutaneous MC38 tumor growth in C57BL6 mice dosed with MCTN‐IRF5 or vehicle. *n* = 3 mice per group (B and C) and *n =* 8 mice per group (D). For B and C bars indicate mean values ± SEM. For D plotted points are the mean ± SEM. Statistics evaluated by two‐tailed unpaired t‐tests (B and C) and two‐way ANOVA with Geisser‐Greenhouse correction (D). ^*^ *p* < 0.05, ^**^ *p* < 0.01.

To investigate the impact of MCTN‐IRF5 treatment on the composition of the TME, we analyzed tumors extracted from a syngeneic MC38 colorectal cancer model. These tumors were collected from mice treated with vehicle and MCTN‐IRF5 and characterized using flow cytometry (**Figure** **6**A; Figures S12 and S13, Supporting Information). This analysis revealed that the MCTN‐IRF5‐treated group of mice exhibited a significantly lower average proportion of macrophages (CD45+CD11b+Ly6G‐Ly6C‐CD170‐F4/80+) within the TME compared with the vehicle control at 18.5% compared with 31.1% of MCs (CD45+CD11b+; Figure 6B). This difference was mirrored by a drop in immunosuppressive (CD45+CD11b+Ly6G‐Ly6C‐CD170‐F4/80+MHCII‐CD206+) TAMs, which composed an average of 10.2% of the MCs for MCTN‐IRF5‐treated mice compared with 15.4% for the vehicle control group (Figure 6B). We also observed that MCTN‐IRF5 treatment increased the proportion of proinflammatory monocytes (CD45+CD11b+Ly6G‐Ly6C+) within the TME to an average of 61.1% of MCs compared with 47.0% in the control group (Figure S14A, Supporting Information). IRF5 therapy has previously been found to increase the proportion of proinflammatory monocytes and decrease the abundance of immunosuppressive tumor‐associated macrophages.^[^ ^20^, ^51^ ^]^

When examining TILs, we found that the average proportion of cytotoxic CD8+ T‐cells (CD45+CD3+CD8+) was significantly higher at 42.3% from MCTN‐IRF5‐treated mice as a percentage of CD45+ lymphocytes compared with 16.7% in the control group (Figure 6C). Improved tumor infiltration of CD8+ T‐cells has been linked to improved prognostic outcomes, suggesting these results are therapeutically relevant.^[^ ^52^ ^]^ Our findings are consistent with previous studies indicating that IRF5 therapy increases CD8+ T‐cell tumor infiltration.^[^ ^20^, ^51^ ^]^ We also observed that the B‐cell proportion of TILs was lower in the MCTN‐IRF5‐treated group, comprising an average of 1.25% of TILs, compared with 11.9% for the vehicle group (Figure 6C). B‐cells were previously implicated as immunosuppressive within the MC38 tumor microenvironment, and removal of these cells suppressed tumor growth, suggesting the B‐cell reduction triggered by MCTN‐IRF5 treatment may impact tumor progression.^[^ ^53^, ^54^ ^]^ Similarly, MCTN‐IRF5 treatment appears to reduce the TIL fraction of CD4+ T‐cells to 8.23% down from 17.4% in the control group (Figure S14B, Supporting Information). CD4+ T‐cell depletion has previously been found to reduce the expansion rate of MC38 tumors and improve responsiveness to checkpoint inhibitor therapy, suggesting that the MCTN‐IRF5‐associated depletion in CD4+ T‐cells we observe may have therapeutic significance.^[^ ^55^, ^56^ ^]^

We examined the efficacy of MCTN‐IRF5 treatment against colorectal cancer by tracking tumor growth in MC38 burdened mice. Mice treated with MCTN‐IRF5 showed significant tumor growth suppression compared to the control group (Figure 6D, Supporting Information). This is consistent with the TME changes we observed following MCTN‐IRF5 treatment, which shifted the TME toward a “hot” state associated with reduced immunosuppression and increased infiltration of effector cells such as CD8+ T‐cells. Indeed, supporting a potential role of CD8+ T‐cells in mediating MCTN‐IRF5 tumor suppression, CD8+ T‐cell depletion has been found to significantly reduce the anti‐tumor activity of IRF5 therapy.^[^ ^20^ ^]^ By shifting the TME from an immunologically “cold” to “hot” state, MCTN‐IRF5 treatment may improve recognition and rejection of tumor cells by the immune system.^[^ ^52^ ^]^

## 3 Conclusion

We have developed a MCTN that selectively targets MCs of the spleen and the TME, while de‐targeting the liver, when administered systemically. Targeting MCs specifically can significantly enhance the effectiveness of immunotherapy. For example, this approach holds the promise of developing more potent cancer vaccines. mRNA is an ideal platform for creating these vaccines, as it can be precisely programmed to express any antigen of interest. Currently, mRNA‐based cancer vaccines, such as autogene cevumeran and mRNA‐4157, have shown promising results in clinical trials. For instance, in a phase 1 study, 6 out of 16 patients with aggressive pancreatic cancer exhibited a tumor‐specific T‐cell response, with no signs of cancer recurrence for 36 months following treatment with autogene cevumeran.^[^ ^57^ ^]^ This vaccine utilizes LPX as a carrier for the mRNA, demonstrating the potential of this approach in improving patient outcomes. Direct dose‐matched comparisons to the cutting‐edge LPX system suggest MCTN could be used at much lower doses due to superior payload delivery, further reducing the barriers to scaling production. Unlike other nanocarriers, which rely on mannose and/or antibody conjugation for MC targeting, which poses challenges in terms of conjugate yield and production cost, MCTN is comprised of a peptide dendrimer, lipids, and a nucleic acid payload, providing a much simpler and more scalable manufacturing pathway.^[^ ^20^, ^21^, ^58^, ^59^ ^]^

We demonstrated that MCTN can be used to induce strong antigen‐specific T‐cell responses by delivering mRNA encoding full‐length protein antigens, rather than short epitopes, and suppress tumor growth.^[^ ^60^ ^]^ Compared with epitope‐based vaccines, which contain small fractions of target antigen that are presented by specific HLA‐haplotypes, vaccines utilizing full‐length antigens can be used in patients with a wider range of HLA‐haplotypes as they incorporate a much broader spectrum of possible epitope combinations.^[^ ^61^ ^]^ Other advantages mRNA vaccines possess over vaccine modalities include serving as a non‐viral vector, avoiding potential safety concerns associated with viral vectors, streamlined production, avoiding the use of expensive cell culture systems, and potentially superior efficacy to induce antigen‐specific immune responses and tumor rejection.^[^ ^60^, ^62^ ^]^

We highlighted the therapeutic potential of directly targeting the MCs within the TME with MCTN through systemic administration using a constitutively active form of proinflammatory IRF5. In vitro, MCTN‐IRF5 treatment downregulated the immunosuppressive activities of macrophages. This mirrored in vivo treatment with a depletion in immunosuppressive TAMs and B‐cells concurrent with increased CD8+ T‐cell infiltration within the TME. This suggests MCTN‐IRF5 treatment shifts tumors from an immunologically “cold” to “hot” phenotype.^[^ ^34^ ^]^ We subsequently demonstrated that MCTN‐IRF5 treatment suppressed colorectal tumor growth. Other research groups have explored delivering RNA directly into tumors via intratumoral injection, demonstrating antitumor efficacy.^[^ ^63^, ^64^ ^]^ However, this approach can be challenging to implement for tumors located deep within the body or those that have metastasized to multiple sites, where direct access is more difficult. Collectively, this study highlights MCTN as a novel immunotherapy, providing an effective platform for both cancer vaccine delivery and direct therapeutic targeting of the TME.

## 4 Experimental Section

### mRNA Sourcing and In‐House Production

5‐methoxyuridine base modified eGFP encoding mRNA was sourced from Trilink Biotechnologies (L‐7201) while N1‐methylpseudouridine base modified mRNA encoding eGFP was custom‐made by Tebubio. Luciferase encoding mRNA incorporating 5‐methoxyuridine modified bases was sourced from Trilink Biotechnologies (L‐7202). Alexa Fluor 488 (AF488) conjugated mRNA contained unmodified bases and was synthesized by RiboPro. mRNA encoding ovalbumin (OVA) contained unmodified bases and was both sourced commercially from Trilink Biotechnologies (L‐7610) and synthesized in‐house (see below). The mRNA encoding constitutively active IRF5 incorporated N1‐methylpseudouridine‐modified bases and was custom‐made by Tebubio. All mRNA sequences incorporated CleanCap.

Regarding in‐house mRNA production, plasmids used as templates for in vitro transcription (IVT) were linearised after the polyA sequence with BspQI (R0712, New England Biolabs). Linearised plasmids were first purified via silica columns (T1130, New England Biolabs), then further purified by precipitation with 3 m sodium acetate, pH 5.2 (15470217, Thermo Fisher) and absolute ethanol (51976, Merck). The linearised plasmid pellet was washed 3 times with 70% ethanol before being dissolved in nuclease‐free water and stored at −80 °C until use. In vitro transcription was performed using the HiScribe T7 High Yield synthesis kit (E2040, New England Biolabs), and capping was performed co‐transcriptionally by addition of Cleancap Reagent AG (3′ OMe) (N‐7413, Trilink Biotechnologies). Following IVT template, DNA was digested by DNAseI treatment (M0303, New England Biolabs) and RNA was purified via silica column (T2040, New England Biolabs), and concentration was measured by absorbance.

### MCTN, LNP, and LPX Formulation

For the MCTN formulations, the peptide dendrimer was diluted in water and HEPES buffer (pH 7.4) to a N:P ratio of 0.6. The desired N:P ratio of 0.6 (ratio of cationic moieties on peptide dendrimer to phosphate groups on mRNA) assumes each peptide dendrimer to contain 10 cationic moieties (6 from arginine residues and 4 from N‐terminal amines). To this, a solution of mRNA diluted in water and HEPES buffer (pH 7.4) was added, mixed, and allowed to incubate for 10 min at room temperature. Next, a liposome dispersion (DOTMA/DOPE 1:1 molar ratio), diluted in water and HEPES buffer (pH 7.4) to achieve the desired mass ratio of lipid to mRNA was added, mixed, and allowed to incubate for at least 10 min at room temperature. Final dilutions of all MCTN formulations were prepared using 25 mm HEPES buffer.

The LNP formulations were prepared using protocols adapted from the literature.^[^ ^65^, ^66^ ^]^ To prepare the LNP, solutions in ethanol were combined to achieve the desired molar ratio SM‐102:DSPC:Cholesterol:DMG‐PEG2k 50:10:38.5:1.5. The aqueous phase contained luciferase mRNA in 10 mm Sodium Citrate buffer (pH 3). The lipid solution was added to the mRNA solution in a 1:3 mixing volume ratio and mixed using a pipette.

The spleen‐targeting LPX‐luciferase formulations were made using protocols based on published precedent.^[^ ^14^ ^]^ RNA‐LPX solutions were diluted to final concentrations in 150 mm NaCl buffer.

Particle size and polydispersity index (Dynamic Light Scattering, DLS) were measured on a Zetasizer Advance Series – Pro (Malvern Panalytical Ltd, Malvern, UK) according to the manufacturer's instructions. Encapsulation efficiency was determined by incubating nanoparticles with RiboGreen reagent for 15 min and measuring fluorescence (excitation/emission wavelengths 485/525 nm) corresponding to unencapsulated mRNA.

### Cell Culture

J774 cells (Sigma; 85011428) were cultured in DMEM supplemented with 2 mm GlutaMAX and 10% heat‐inactivated FBS. When 80% confluent J774 cells were passaged using cell scrapers and reseeded at a density of 300 000 cells/ml. For transfection, 100 000 cells/well were seeded into a 96‐well format.

Murine bone marrow‐derived monocytes were isolated using the STEMCELL Tech EasySep Mouse Monocyte Isolation Kit (STEMCELL Technologies; 19861) from bone marrow extracts from Balb/c mice. Prior to monocyte isolation, bone marrow extracts from 3–4 mice were pooled together to create independent biological samples for testing. Monocytes were differentiated by 6‐day culture in complete DMEM media (enriched with 1% GlutaMAX, 1% pen/strep, and 10% heat‐inactivated FBS; Gibco) supplemented with 50 ng mL^−1^ murine M‐CSF (Peprotech). For the last 2 days of the differentiation, the macrophages were activated with the addition of 20 ng mL^−1^ IL‐4 (Peprotech) to the media (complete activation media). Adherent differentiated macrophages were harvested using 5 mm EDTA (Invitrogen) and seeded into 12‐well plates (2.4 × 10^5^ cells per well) for the immunophenotyping and RNA‐seq experiments and 24‐well plates (2.5 × 10^5^ cells per well) for the cytokine secretion and IRF5 protein quantification experiments.

Cryovials of human monocytes sourced from STEMCELL Technologies (70035.1) and Research Donors Ltd. (MONM05‐14P) were thawed, diluted in ImmunoCult‐SF Macrophage Medium (STEMCELL Technologies; 10961), and spun down at 300 g for 10 min. Cells were cultured in ImmunoCult‐SF Macrophage Medium supplemented with 50 ng mL^−1^ human M‐CSF (Peprotech), henceforth known as ImmunoCult, for 4 days. Human macrophages were subsequently activated by a half media exchange, supplementing ImmunoCult with 10 ng mL^−1^ (end concentration) human IL‐4 (Peprotech) and replacement human M‐CSF (end concentration 50 ng/ml) for 2 days. Cells were prepared and transfected in the same manner as described for the BMDMs, except using IL‐4‐supplemented ImmunoCult media instead of DMEM‐based complete activation media.

All primary human monocyte samples (STEMCELL Technologies and Research Donors Ltd.) were acquired with Research Ethics Committee (REC; 20/LO/0325) and Institutional Review Board (IRB) approval and were anonymized.

### Bone Marrow Isolation

For the isolation of primary murine monocytes, bone marrow was extracted from the femurs and tibias of Balb/c mice. Briefly, after sacrifice, the femurs and tibias were removed from each mouse. These bones were then sterilized using 70% ethanol. Using a syringe and 25G needle bone marrow cells were flushed out of the bones using RPMI supplemented with 10% heat‐inactivated fetal bovine serum (FBS) and 1% pen/strep. The flowthrough was filtered through 70 µm filters and subsequently used for monocyte isolation. This work was conducted by Epistem (Manchester, UK).

### In Vitro Transfection Procedure

For all experiments, MCTN formulations were prediluted in 25 mm HEPES buffer prior to addition to cells, whilst LNP and LPX formulations were diluted in 20 mm Tris buffer and 150 mm NaCl buffer, respectively. For the immunophenotyping, RNA‐seq, and cytokine secretion assays, macrophages were transfected overnight with 3 µg mRNA/well of the test formulations added to complete activation media (mouse) or IL‐4‐supplemented ImmunoCult (human). For the in vitro luciferase assays, J774 cells were preincubated with or without inhibitors for 30 min prior to transfection for 1 h with 0.33 µg mRNA/well with or without inhibitors, followed by a wash step and full media exchange with or without inhibitors. For the IRF5 protein quantification experiments, macrophages were transfected for 1 h with 2 µg mRNA/well of the test formulation in serum‐free media, followed by a full media exchange with complete activation media. Untransfected control consisted of 25 mm HEPES, and the transfection control was a luciferase payload (L‐7202; TriLink Biotechnologies). Between the addition of each test condition plates were shaken back and forth to ensure mixing.

### In Vitro Luciferase Assays

J774 cells were either left untreated or treated with 125 µm cytochalasin D (STEMCELL Technologies 100–0556) to target phagocytosis and macropinocytosis, 25 µm rottlerin (Tocris 1610) to target macropinocytosis, or 0.5 µm bafilomycin A1 (Enzo Life Sciences BML‐CM110) to inhibit endosomal acidification for 30 min prior to transfection. All inhibitors were reconstituted in DMSO prior to use. Following this J774 cell formulations were added directly to the cells with or without additional inhibitors and J774 cells were transfected for 1 h. For the cytochalasin D and rottlerin‐treated cells, the transfection was followed by a wash step and full media exchange with J774 culture media containing no inhibitors. For the bafilomycin A1‐treated cells, the transfection was followed by a wash step and full media exchange with J774 media containing 0.5 µM bafilomycin A1. Cells were left 4 h in a humidified 37 °C incubator with 5% CO_2_. Cells were then washed using PBS and lysed using reporter lysis buffer, and the resulting lysates were analyzed for luciferase activity as per manufacturer's instructions (Luciferase Assay System kit from Promega; E4030). Total protein content for each lysate sample was quantified the BioRad Protein Assay Kit (5000001) as per the manufacturer's instructions. Luciferase activity was calculated by normalizing the luminescence measured for each sample in relative light units (RLU) by the total protein content (in mg) of each sample. To calculate % inhibition induced by the pharmacological agents, the luciferase activity of each test sample was compared against corresponding control samples (cells transfected in the absence of any inhibitor).

### In Vivo Studies

All animal experiments were reviewed and approved by the local Animal Welfare and Ethical Review Board (AWERB) and approved by the UK Home Office in accordance with the Animals Act 1986 and the ARRIVE guidelines (EC2021‐184, P7E32C4C0/1 and PP0969406).

Animals were housed and maintained in rooms under controlled conditions of temperature (19–23 °C) and humidity (55% ± 10%), photoperiod (12 h light/12 h dark), and air exchange, with food and water provided ad libitum. The facilities had been approved by the Home Office and meet all current regulations and standards of the UK (The Animals (Scientific Procedures) Act 1986).

### In Vivo Biodistribution Studies

The in vivo biodistribution study in wild‐type mice was carried out on female CD‐1 mice aged between 6–8 weeks, which had been left to habituate for 1 week upon arrival at the animal facility. These mice were injected intravenously via the tail vein with either MCTN‐luciferase, LPX‐luciferase or unencapsulated luciferase mRNA at 0.75 mg kg^−1^. For the in vivo biodistribution in MC38 tumor‐burdened mice, the model was generated by inoculating 2 × 10^6^ MC38 cells subcutaneously on the flank of 8 weeks old female C57BL6 mice. The mice were administered intravenously with either MCTN‐luciferase or unencapsulated luciferase mRNA at 2 mg kg^−1^ once tumors reached a size of 200 mm^3^. 6 h after treatment, bioluminescence imaging with the MS Lumina II (Perkin Elmer) imaging system was used to measure live luciferase signal within each mouse following intraperitoneal dosing with 150 mg kg^−1^ D‐luciferin (Perkin Elmer). Mice were anesthetized using 5% isoflurane and placed on a heated imaging platform and maintained on 2.5% isoflurane. Images were taken 10 min after D‐luciferin administration with an exposure time of 45 s. For ex vivo bioluminescence imaging, mice were sacrificed, and liver and spleen were extracted. These isolated organs were then placed in separate wells in a 24‐well plate containing 0.3 mg mL^−1^ D‐luciferin diluted in PBS and subsequently imaged. The bioluminescence signal was quantified by measuring photon flux (photon/s) using the Living Image software (Perkin Elmer) within defined regions of interest.

### In Vivo Uptake Study

6‐week‐old female C57BL6 mice were implanted with 1 × 10^7^ MC38 cells each and randomized into separate treatment groups when the mean tumor volume reached 500 mm^3^. Mice were administered intravenously with 2.25 mg kg^−1^ of either MCTN encapsulated or unencapsulated AF488‐mRNA, via the tail vein. 4 h post‐treatment, mice were culled and the tumors and spleens extracted for flow analysis. Tumors were digested using a tumor dissociation kit and gentleMACS Octo dissociator (Miltenyi). Spleens were pushed through a 70 µm cell strainer using a syringe plunge to dissociate the tissue into a cell suspension. The splenic cell suspensions were then treated with Red Blood Cell Lysing Buffer HYBRI‐MAX (Merck) prior to antibody staining. Single cell suspensions were stained as described below and analyzed using the Attune NxT flow cytometer (ThermoFisher).

### In Vivo Assessment of Vaccine Efficacy

For the ELISpot assays, 7–8 week‐old female Balb/c mice were treated intravenously (via tail vein) 3 times with 2 mg kg^−1^ MCTN‐OVA or vehicle (25 mm HEPES buffer) at weekly intervals. 7 days after the final vaccination, mice were sacrificed and spleens isolated. Splenocytes were isolated by dissecting and gently stroking spleens in splenocyte culture media (RPMI with 10% heat‐inactivated FBS, 1% pen/strep, and 10 mm HEPES buffer). After resuspension cells were passed through 70 µm filters. Cells were spun down at 800 g for 3 min and then resuspended in culture media for cell counting.

For the tumor growth suppression model, 5–6 week‐old female C57BL6 mice were vaccinated using the same schedule as described above for the Balb/c mice with 2 mg kg^−1^ MCTN‐OVA or MCTN‐eGFP. 6 days after the final vaccination, mice were inoculated subcutaneously into the with right rear flank with 200000 B16F10‐OVA cells. Palpable tumor growth was measured using calipers at 2–3 day intervals. Length (*L*) and width (*W*) measurements were taken of the tumors. Tumor volume (*V*) in all experiments was estimated using the following equation: $V=\frac{L*W^{2}}{2}\;$. For all experiments, it was predetermined that a humane end point would be reached if tumors reached or exceeded 2000 mm^3^ in size.

### ELISpot Assays

The MabTech murine IFN‐γ ELISpot assay (3321‐4AST‐10) was used to examine splenocyte immune responses to MCTN‐OVA treatment. 250 000 splenocytes were seeded per well (100 000 for the positive control condition) in precoated 96‐well plates in a total of 75 µL splenocyte culture media (RPMI with 10% heat‐inactivated FBS, 1% pen/strep, and 10 mm HEPES buffer). Protein stimuli were prepared as 2x concentration stocks from pre‐aliquoted master stocks. 75 µl of these 2x concentrated stocks of protein stimuli were added to each well as appropriate to achieve a final concentration of 2 µg/ml. Prior to dilution in splenocyte culture media, ovalbumen protein (Sigma) was dissolved in water, while full‐length KLK2 protein (irrelevant control; Biotechne) was resuspended in sodium citrate and sodium chloride. Negative controls were produced by adding 75 µl/well splenocyte cell culture media without stimuli additives. The positive control contained a final 1:1000 dilution of Cell Activation Cocktail stock (without Brefeldin A; Biolegend), which contains 25 µg ml^−1^ phorbol‐12‐myristate 13‐acetate (PMA) and 500 µg ml^−1^ ionomycin dissolved in DMSO. After the addition of stimuli, splenocytes were left in a humidified 37 °C incubator with 5% CO_2_ for 2 days. After this period, the spots were developed as per the manufacturer's instructions, imaged, and quantified using the IRIS 2 ELISpot reader in conjunction with the Mabtech Apex analysis software (MabTech). For data presentation, spot counts were adjusted for 1 million splenocytes per well.

### Examination of MCTN‐IRF5 Impact on the MC38 Syngeneic Tumor Model

For the in vivo examination of the impact of MCTN‐IRF5, the syngeneic subcutaneous MC38 tumor model was used. 8‐week‐old female C57BL6 mice were inoculated in the flank with 250000 MC38 cells per mouse. When an average tumor volume of 50–100 mm^3^ was reached, as determined by caliper measurements, mice were randomized and treated intravenously via the tail vein with either vehicle (25 mM HEPES buffer) or MCTN‐IRF5. Mice were subsequently re‐dosed at 2–3 day intervals with vehicle or MCTN‐IRF5 as appropriate. For the flow cytometry analysis, mice were culled 9 days after treatment initiation and tumors isolated. Tumors were subsequently dissociated with a tumor dissociation kit and the GentleMACS dissociator (Miltenyi). These tumor cell suspensions were stained and immunophenotyped by flow cytometry analysis. For the tumor growth experiment, tumors were measured at 2–3 day intervals using caliper measurements to determine tumor volume. In vivo work for this study was conducted in the AAALAC accredited and SPF facilities of InnoSer Laboratories, Diepenbeek, Belgium.

### Cell Staining and Flow Cytometry

Cells were washed with PBS and collected using 5 mm EDTA and mechanical scraping into separate tubes. Cells were spun down at 400 g for 5 min and resuspended in 100 µl/sample fixable Live/Dead Aqua stain (diluted in PBS) and left in the fridge for 30 min. Following further centrifugation, disposal of the live/dead stain and a PBS was, cells were blocked with 50 µl/well of a 2x block solution containing FcX block (1:50) for mouse or TruStain Human FcX block diluted in FACS buffer (PBS with 2 mm EDTA and 0.5% w/v BSA) for 5 min in the fridge. After blocking, 50 µl/well 2x extracellular staining buffer was added to each sample and incubated for 30 min in the fridge. The following antibodies used to label mouse BMDMs were Cd11b‐APC (Biolegend; 101212) and CD80‐PE (Biolegend;104708). Prior to flow cytometry analysis, cells were fixed using 4% PFA. Fold changes in CD80 surface expression by BMDMs post‐treatment with MCTN‐IRF5 or MCTN‐luciferase were calculated by dividing the CD80 channel median fluorescence intensity (MFI) of the live BMDMs (CD11b+) treated with MCTN‐IRF5/MCTN‐luciferase by the CD80 channel MFI of the untreated BMDMs. Human macrophages were labelled with CD11b‐APC (Biolegend; 101212), CD14‐PE (Biolegend; 325606) and CD80‐BV711 (Biolegend; 305236). eGFP transfection was calculated on live macrophages (CD14+CD11b+ cells).

Compensation samples were produced by staining compensation beads (Arc beads for the live/dead stain, UltraComp Plus beads for the antibodies, and GFP beads for the eGFP transfected cells). Following centrifugation and a wash with FACS buffer, all samples were fixed with 4% PFA for 10 min. Post‐fixation excess PFA solution was washed away with FACS buffer, and samples were resuspended in FACS buffer and filtered for flow cytometry analysis. Samples were run on a BD Fortessa Flow Cytometer.

For the staining of tumor cells from the in vivo uptake study, cells were processed as described above with the eFluor506 fixable viability dye and with a mixture of antibodies labelling MCs, including: CD45‐PE‐Cyanine5.5, CD11b‐PE, CD11c‐PE‐Texas‐Red, Ly6G‐APC‐eFluor‐780, Ly6C‐PerCP‐Cyanine5.5, and F4/80‐AF647. Uptake of AF488‐mRNA was detected by examining fluorescence in the eGFP channel.

For the staining of splenic cells from the in vivo uptake study, cells were stained in two separate panels one for MCs and another for lymphocytes. For both panels, cells were stained as above with the eFluor506 fixable viability dye. For the MC panel, cells were stained with a mixture of antibodies, including: CD45‐PE‐Cy5.5, CD11b‐PE, CD11c‐PE‐Texas‐Red, Ly6G‐APC‐eFluor‐780, Ly6C‐PerCP‐Cyanine5.5, F4/80‐AF647, and CD19‐SuperBright‐702. For the lymphocyte panel cells were stained with a mixture of antibodies, including: CD45‐SuperBright‐600, CD3‐AF700, CD4‐BV711, CD8‐PE‐Cy7, and NKp46‐PE‐Cy5.5.

For the staining of tumor cell suspensions from the MCTN‐IRF5 study, tumor samples were divided into two different antibody staining panels, a MC panel and a lymphocyte panel, and stained in 2 million cell cohorts. Both panels incorporated the live/dead dye Fixable Viability Stain 575 V (BD Horizon). All antibodies were from Miltenyi unless indicated otherwise. The MC panel included these antibodies: CD45‐APC‐Vio770, CD11b‐Viogreen, F4/80‐PE‐Vio‐770, MHCII‐APC, CD206‐AF488 (BD Biosciences), Ly6G‐REA526, Ly6C‐REA796 and CD170‐PE‐CF594 (BD Biosciences). The lymphocyte cell panel included these antibodies: CD45‐APC‐Vio770, CD19‐PEVio615, CD3‐FITC, CD4‐VioBlue, CD8‐VioGreen, and NKp46‐PE. Post‐staining cells were analyzed using the MACSQuant Analyzer 16 (Miltenyi).

### RNA‐Seq Sample Preparation and Sequencing

20 h post‐transfection, cells were washed with PBS and lysed with 500 µl/sample RNAqeous lysis buffer, scraped and collected. RNA was extracted using the RNAqeous total RNA Isolation kit as per the manufacturer's instructions (ThermoFisher; AM1912). Total RNA samples were subsequently stored in a ‐80 °C freezer prior to submission for RNA‐seq by Imperial BRC Genomics Facility for library preparation and sequencing. Samples were sequenced by the NextSeq 2000 with 25 m reads used per sample.

### RNA Sequencing and Analysis

RNA sequencing (RNA‐seq) was performed to assess transcriptional changes induced by the therapeutic mRNA payloads. Libraries were sequenced on an Illumina NextSeq 2000 using a P3 flow cell for 200 cycles, generating paired‐end 100 bp reads. A custom reference genome was built with STAR (v2.7.10a) using the GRCm39 mouse reference genome (GCF_000001635.27), supplemented with sequences for the therapeutic constructs (luciferase and IRF5), and annotations from the corresponding GTF file.^[^ ^67^ ^]^ STAR was run in single‐pass mode with –quantMode GeneCounts enabled to obtain gene‐level read counts.

Differential expression analysis was performed using DESeq2 (v1.40.2), with log2 fold change shrinkage applied using the normal method.^[^ ^68^ ^]^ Genes corresponding to the transgenes were excluded from the differential expression analysis. Wald test *P*‐values were adjusted using the Benjamini–Hochberg method. Data visualization, including volcano plots, was performed in R (v4.3.1) using Bioconductor (v3.17) packages.

### Cytokine Secretion Assay

After overnight transfection, the transfection media was exchanged with 500 µl/well complete activation media. 24 h later the media was harvested, spun at 400 g for 10 min, and the sample supernatants stored in the ‐80 °C freezer. Meanwhile the cells were washed with PBS and lysed with 200 µl/well RIPA buffer for 5 min on a shaker (400 rpm). Lysates were spun at 400 g for 10 min and supernatants stored in a ‐80 °C freezer. Supernatants were later analyzed for murine cytokine content (IL12p40) using the LEGENDplex mouse macrophage/microglia panel (Biolegend; 740845) and human cytokine content (IL12p70 and TNF‐α) using the LEGENDplex human macrophage/microglia panel (Biolegend; 740503) as per the manufacturer's instructions. Lysates were analyzed using the Pierce BCA assay (ThermoFisher; 23225) to determine total protein content of each well. The cytokine content per well was subsequently normalized by total protein content to take account of potential differences in cell density between wells.

### IRF5 Protein Quantification

At appropriate time points cells were washed with PBS. After the PBS wash was discarded cells were lysed with 200 µl/well RIPA buffer supplemented with 1:100 Halt protease and phosphatase inhibitor cocktail (ThermoFisher; 78440) for 5 min on a plate shaker (400 rpm). Lysates were harvested and spun at 400 g for 10 min. Lysate supernatants were stored in a −80 °C freezer prior to analysis. Lysate IRF5 content was evaluated using IRF5 ELISA kits (Abcam; ab282878). A Pierce BCA assay was run separately to determine the total protein content of each well. IRF5 content per well was normalized by the total protein content of each well.

### Statistical Analysis

FlowJo was used to analyze the flow cytometry data, including gating, generating plots, and calculating sample statistics. GraphPad Prism was used for statistical analyses and generating histograms and scatter plot figures. Data are presented as mean ± SEM. Single‐sample t‐tests were used to test whether luciferase signal inhibition following transfection of J774 cells in the presence of cytochalasin D, rottlerin, or bafilomycin A1 was significant compared against a hypothetical mean of 0% inhibition. For statistical comparison of independent samples, unpaired two‐tailed t‐tests or Mann‐Whitney tests were used. For paired datasets, statistics were evaluated using two‐tailed ratio paired t‐tests. For multiple group comparisons, one‐way ANOVA with Tukey's post‐hoc test was used. The two‐way mixed‐effects model or two‐way ANOVA using the Geisser‐Greenhouse correction was used to statistically evaluate the impact of treatment condition on tumor growth (OVA‐B16F10 and MC38 tumor models). For the RNA‐seq data, differential expression analysis was performed using DESeq2 (v1.40.2) utilizing the Wald test and *P*‐value adjustment by the Benjamini–Hochberg method. ^*^ *p* < 0.05, ^**^ *p* < 0.01 and ^***^ *p* < 0.001 were considered statistically significant, with *p* < 0.05 as the threshold for significance.

## Conflict of Interest

All authors are employees of Nuntius Therapeutics Limited, of which A.K. and B.N. are co‐founders and directors. This work is associated with patent application PCT/EP2023/071607.

## Author Contributions

L.J.B. project administration, conceptualization, experiment execution, data analysis, curation, and manuscript writing. L.M. experiment execution, data analysis, project administration. T.H.B. Data analysis. P.S.: experiment execution, data analysis, project administration. A. Leach: experiment execution, project administration. P.K., M.A., A.L. experiment execution. B.N. Project initiation, funding acquisition. A.K. Project initiation, funding acquisition, project administration, conceptualization, data analysis, manuscript writing, supervision.

## Supporting information



Supporting Information

Supporting Information

## Data Availability

The data that support the findings of this study are available from the corresponding author upon reasonable request.
