# Supplementary material for: Precision Targeting of Myeloid Cells via Peptide Dendrimer‐Lipid Nanocarriers: A Novel Platform for Potent Cancer Immunotherapies
Source: Adv Sci (Weinh). 2025 Sep 19;12(46):e14417. doi: 10.1002/advs.202514417 (PMC12697770; doi:10.1002/advs.202514417)
Supplement: Supplementary file 1 — Supporting Information [file ADVS-12-e14417-s001.docx]

**Supporting Information:**

**Constitutively active IRF5 amino acid sequence:**

MNQSIPVAPTPPRRVRLKPWLVAQVNSCQYPGLQWVNGEKKLFCIPWRHATRHGPSQDGDNTIFKAWAKETGKYTEGVDEADPAKWKANLRCALNKSRDFRLIYDGPRDMPPQPYKIYEVCSNGPAPTDSQPPEDYSFGAGEEEEEEEELQRMLPSLSLTEDVKWPPTLQPPTLQPPVVLGPPAPDPSPLAPPPGNPAGFRELLSEVLEPGPLPASLPPAGEQLLPDLLISPHMLPLTDLEIKFQYRGRPPRALTISNPHGCRLFYSQLEATQEQVELFGPISLEQVRFPSPEDIPSDKQRFYTNQLLDVLDRGLILQLQGQDLYAIRLCQCKVFWSGPCASAHDSCPNPIQREVKTKLFSLEHFLNELILFQKGQTNTPPPFEIFFCFGEEWPDRKPREKKLITVQVVPVAARLLLEMFSGELDWDADDIRLQIDNPDLKDRMVEQFKELHHIWQSQQRLQPVAQAPPGAGLGVGQGPWPMHPAGMQ

Sequence based on UniProt: Q13568-4. Serine to aspartate point mutations are highlighted in blue.

Figure S1: Chemical structure of the peptide dendrimer (**A**) and lipid (**B**) components of MCTN. The peptide dendrimer component of MCTN is formed of 2 generations with the amino acid sequence (RHL)4(KRHL)2KGSC-NH2 and an associated molecular mass of 3087.8 g/mol.

Figure S2: Gating strategy for the immunophenotyping of transfected murine bone marrow-derived macrophages (BMDMs). Example flow cytometry plots are displayed. BMDM were transfected overnight with MCTN-eGFP. For the BMDM phenotyping panel the example given is a vehicle control sample of BMDMs treated with 25 mM HEPES buffer. For detection of eGFP, placement of the eGFP gates was determined using untransfected samples (control) enabling clear detection of transfected cells in MCTN-eGFP treated samples. Percentages indicate gated cells as a proportion of parent population. MFI refers to median fluorescence intensity.

Figure S3: Gating strategy for the human monocyte-derived macrophages. Control refers to macrophages treated with vehicle (25 mM HEPES buffer). Only live macrophages (CD14+CD11b+ cells) were evaluated for eGFP expression. Percentages indicate gated cells as a proportion of parent population.

Figure S4: **A**, Impact of inhibiting phagocytosis and macropinocytosis mediated MCTN-luciferase uptake by macrophages (J774 cells), using cytochalasin D and rottlerin, on resulting luciferase expression. The impact of inhibiting endosomal acidification using bafilomycin A1 on endosomal escape and luciferase expression was also examined. *n*=5 independent experiments with rottlerin and bafilomycin A1 and *n*=4 independent experiments with cytochalasin D. % inhibition was calculated relative to the control group (MCTN-luciferase in the absence of inhibitors). **B**, Comparison of LNP-luciferase, LPX-luciferase and MCTN-luciferase transfection efficiency of murine macrophages (J774 cells) as measured by luciferase activity. The control group was treated with vehicle buffer (25mM HEPES). *n*=4 independent experiments for control and MCTN-luciferase and 3 independent experiments for LNP-luciferase and LPX-luciferase. For all graphs bars indicate mean values ± SEM. Statistics were evaluated using one-sample t-tests with a hypothetical mean of 0% inhibition for **A**. **B** was evaluated using one-way ANOVA with Tukey’s post-hoc test. ***p* < 0.01, ****p* < 0.001.

Figure S5: **A**, Total flux calculated for liver and spleens isolated from MCTN-luciferase treated mice compared. Bioluminescence images of isolated livers and spleens are shown on the right-hand side. Control mice were treated with unencapsulated luciferase mRNA. Liver and spleens isolated from 3 mice. **B**, Comparison of splenic delivery of luciferase mRNA encapsulated by LPX (LPX-luciferase) and MCTN (MCTN-luciferase). Bioluminescence images of isolated spleens are shown on the right-hand side. *n*=3 mice per group. Bars indicate mean values ± SEM. Statistics were evaluated using a two-tailed paired t-test for **A** and a two-tailed unpaired t-test for **B**. ***p* < 0.01, ****p* < 0.001.

Figure S6: Flow cytometry gating strategy for the MCTN uptake study examining splenic cells from mice treated with unencapsulated (control) or MCTN encapsulated AF488-mRNA (MCTN-AF488). Percentages indicate gated cells as a proportion of parent population.

Figure S7: In vivo uptake of Alexa Fluor 488 (AF488) tagged mRNA by splenic cells following intravenous (I.V.) treatment with MCTN encapsulated AF488-mRNA (MCTN-AF488) or unencapsulated AF88-mRNA (control) was detected by flow cytometry (gating strategy in Figure S6). **A**, AF488-uptake by splenic macrophages (CD45+CD11b+CD11c-Ly6G-Ly6C-F4/80+), monocytes (CD45+CD11b+CD11c-Ly6G-Ly6C+F4/80-) and dendritic cells (CD45+CD11b+CD11c+). **B**, AF488-uptake by splenic CD4+ (CD45+CD3+CD4+) T-cells, CD8+ T-cells (CD45+CD3+CD8+) and B-cells (CD45+CD11b-CD19+). Bars indicate mean values ± SEM. Unpaired two-tailed t-tests used to evaluate statistics. ***p* < 0.01, **p* < 0.05.

Figure S8: Images of murine IFN-γ ELISpot wells developed and analyzed for evaluating MCTN-OVA efficacy. Top panel details wells imaged from vehicle (25 mM HEPES buffer) treated Balb/c mice. Lower panel details wells imaged from MCTN-OVA treated Balb/c mice. Each test condition was run in duplicate technical replicates.

Figure S9: Results from IFN-γ ELISpot assays run on splenocytes isolated from female Balb/c mice vaccinated intravenously 3 times at weekly intervals with MCTN-OVA or administered with vehicle. 100,000 splenocytes per well were stimulated with PMA/Ionomycin (positive control) for 2 days. Example ELISpot well images are shown below each condition. *n*=4 mice per group.

Figure S10: Biodistribution of MCTN-luciferase examined in MC38 tumor-burdened C57BL6 mice. **A**, Schematic detailing key stages of the biodistribution study. **B**, Total flux measured in live animals within the tumor (region of interest). *n*=3 mice for control and *n*=2 mice for the MCTN-luciferase group. Bioluminescence images captured from live mice after D-Luciferin treatment are shown on the right. The control group was treated with unencapsulated luciferase mRNA while the MCTN group was treated with MCTN encapsulated luciferase mRNA (MCTN-luciferase).

Figure S11: Flow cytometry gating strategy for the MCTN uptake study in MC38 tumors from mice treated with unencapsulated (control) or MCTN encapsulated AF488-mRNA (MCTN-AF488). Percentages indicate gated cells as a proportion of parent population.

Figure S12: Example gating strategy for determining the macrophage composition of the tumor microenvironment. Plots in the top panel come from vehicle (25 mM HEPES buffer) treated mice and plots in the bottom panel from MCTN-IRF5 treated mice. PM refers to proinflammatory monocytes. Percentages indicate gated cells as a proportion of parent population.

Figure S13: Example gating strategy for tumor-infiltrating lymphocytes (TILs) from digested MC38 tumor samples. The plots in the top panel come from vehicle (25 mM HEPES buffer) treated mice. The plots in the bottom panel come from MCTN-IRF5 treated mice. Percentages indicate gated cells as a proportion of parent population.

Figure S14: Additional immune cell subtypes analyzed by flow cytometry within the tumor microenvironment of MC38 burdened mice. **A**, Proinflammatory monocytes (CD45+CD11b+Ly6G-Ly6C+) and neutrophils (CD45+CD11b+Ly6G+) as a proportion of myeloid cells (CD45+CD11b+). **B**, CD4+ T-cells (CD45+CD3+CD4+) and NK cells (CD45+NKp46+) as a proportion of tumor-infiltrating lymphocytes (TILs; CD45+). *n*=3 mice per group. Statistics evaluated using two-tailed unpaired t-tests. ns – non-significant (*p* > 0.05), **p* < 0.05.
